# An N-terminal domain helical motif of Prototype Foamy Virus Gag with dual functions essential for particle egress and viral infectivity

**DOI:** 10.1186/1742-4690-10-45

**Published:** 2013-04-25

**Authors:** Juliane Reh, Annett Stange, Anne Götz, Marlene Rönitz, Arend Große, Dirk Lindemann

**Affiliations:** 1 Institut für Virologie, Medizinische Fakultät “Carl Gustav Carus”, Technische Universität Dresden, Fetscherstr. 74, 01307, Dresden, Germany; 2 CRTD/DFG-Center for Regenerative Therapies Dresden-Cluster of Excellence, Technische Universität Dresden, Fetscherstr. 105, 01307, Dresden, Germany

**Keywords:** Foamy virus, Budding, Protein-protein Interaction, Helical motif

## Abstract

**Background:**

Foamy viruses (FVs) have developed a unique budding strategy within the retrovirus family. FV release requires co-expression and a highly specific interaction between capsid (Gag) and glycoprotein (Env), which cannot be complemented by heterologous Env proteins. The interaction domain in FV Env has been mapped in greater detail and resides mainly in the N-terminal tip of the cytoplasmic domain of the Env leader peptide subunit. In contrast, the corresponding domain within Gag is less well defined. Previous investigations suggest that it is located within the N-terminal part of the protein.

**Results:**

Here we characterized additional Gag interaction determinants of the prototype FV (PFV) isolate using a combination of particle release, GST pull-down and single cycle infectivity analysis assays. Our results demonstrate that a minimal PFV Gag protein comprising the N-terminal 129 aa was released into the supernatant, whereas proteins lacking this domain failed to do so. Fine mapping of domains within the N-terminus of PFV Gag revealed that the N-terminal 10 aa of PFV Gag were dispensable for viral replication. In contrast, larger deletions or structurally deleterious point mutations in C-terminally adjacent sequences predicted to harbor a helical region abolished particle egress and Gag – Env protein interaction. Pull-down assays, using proteins of mammalian and prokaryotic origin, support the previous hypothesis of a direct interaction of both PFV proteins without requirement for cellular cofactors and suggest a potential direct contact of Env through this N-terminal Gag domain. Furthermore, analysis of point mutants within this domain in context of PFV vector particles indicates additional particle release-independent functions for this structure in viral replication by directly affecting virion infectivity.

**Conclusions:**

Thus, our results demonstrate not only a critical function of an N-terminal PFV Gag motif for the essential capsid - glycoprotein interaction required for virus budding but also point out additional functions that affect virion infectivity.

## Background

Glycoproteins of lipid membrane enveloped viruses harbor the main essential determinants of viral particle tropism. This includes host cell receptor and/or co-receptor recognition as well as the machinery required for fusion of viral and cellular membranes during virion entry. As a consequence enveloped viruses such as retroviruses are highly dependent on successful incorporation of their particular glycoprotein(s) during the budding step of particle morphogenesis in the infected host cell to ensure subsequent productive infection of target cells. For orthoretroviruses different, but related mechanisms of glycoprotein encapsidation into budding virions are apparent. This explains the orthoretroviral capacity for specific incorporation of the cognate glycoprotein but also pseudotyping with glycoproteins from unrelated viral families (reviewed in [1]). Factors, such as co-trafficking or recruitment of capsid and glycoproteins to specific membrane domains, direct or indirect interaction between both proteins, as well as steric compatibility of cytoplasmic glycoprotein domains with specific virus capsids, influence glycoprotein inclusion.

Spuma- or foamy virus (FVs) budding is reported to occur at different cellular membranes/locations [2-4]. All known FV species with the exception of equine FV (EFV) are thought to bud predominantly into intracellular compartments, a phenotype that correlates with an ER retrieval signal located at the C-terminus of the respective glycoproteins [5-7]. FVs are unique among retroviruses, as budding requires co-expression of both the capsid (Gag) and the viral glycoprotein (Env) (reviewed in [8,9]). A very specific interaction of both proteins, presumably taking place at the trans-Golgi network, is necessary for this step in replication since the FV Gag protein lacks a membrane-targeting signal [3,10-12]. The glycoprotein domain targeted by the capsid was mapped to the N-terminus of cytoplasmic domain (CyD) of the FV Env signal- or leader peptide (LP) subunit (gp18^LP^ for prototype FV, PFV) [13,14]. Two evolutionary conserved tryptophan residues (W_10_, W_13_ in PFV Env) of the Env LP CyD are known to be essentially involved in the Gag interaction. This special function of a retroviral glycoprotein signal peptide in virion morphogenesis is the result of the unusual biosynthesis of FV Env proteins that lack cotranslational removal of the signal peptide required for targeting to the secretory pathway [13]. Instead, FV Env proteins are processed post-translationally, late during cell surface transport, giving rise to a glycoprotein complex in released FV particles composed of a LP subunit with type II membrane orientation, an extracellular surface (SU) subunit (gp80^SU^ for PFV), and a type I membrane topology transmembrane (TM) subunit (gp48™ for PFV) [13-16].

**Figure 1:**
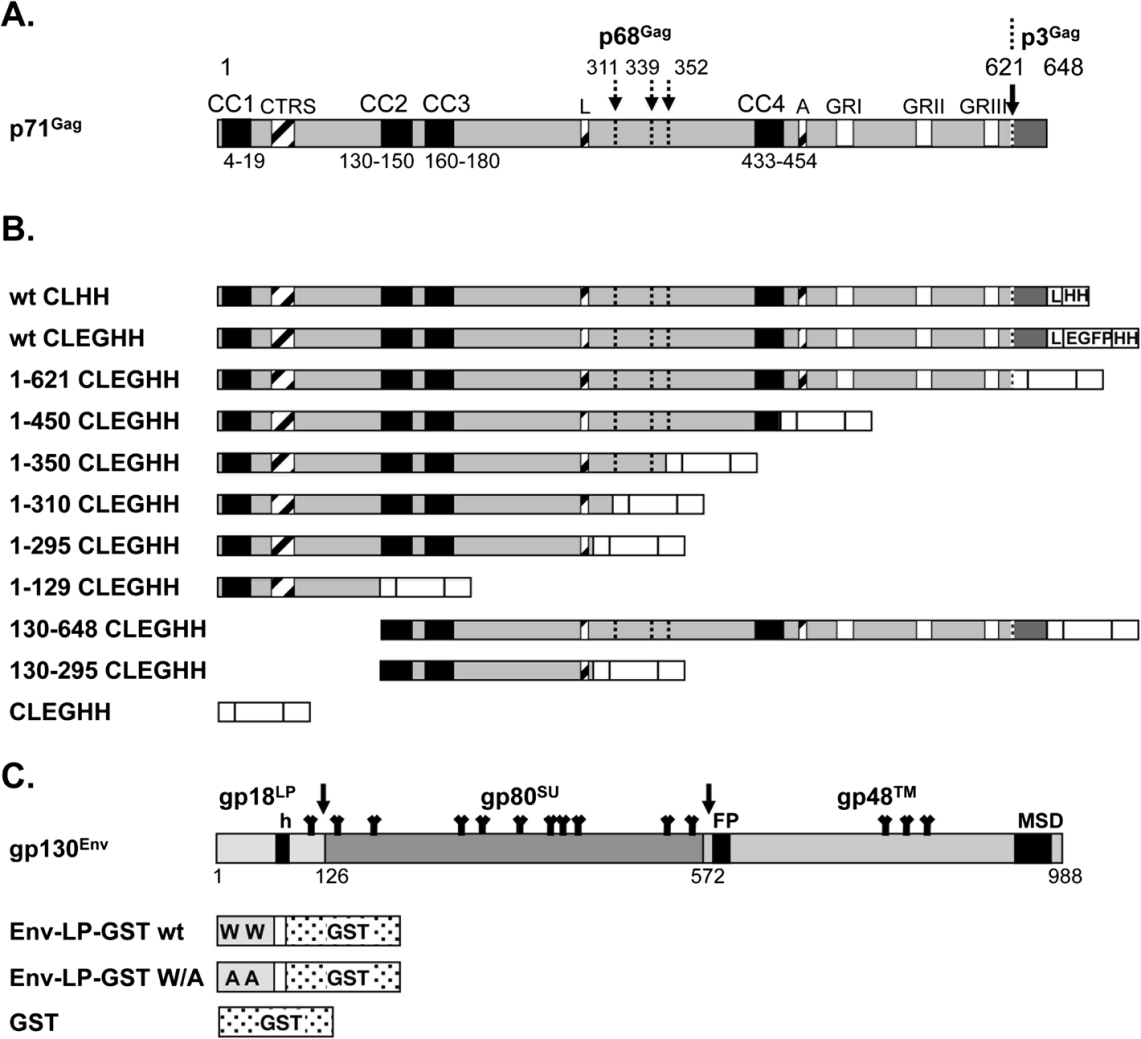
**Schematic illustration of the mammalian Gag and Env expression constructs.** (**A**) Schematic outline of the PFV Gag precursor protein. Structural and functional domains are indicated in differentially shaded boxes as explained below. The major processing site in p71^Gag^ is marked by a solid arrow and potential secondary cleavage sites by dashed arrows. Numbers indicate amino acid positions in PFV Gag. (**B**) Schematic outline of the PFV Gag expression constructs. All constructs harbor a C-terminal flexible (Gly_4_Ser_1_)_3_ linker (L) and either a HA-6xHis-tag (HH) or EGFP-HA-6xHis-tag (EGFP-HH) indicated as white boxes. (**C**) Schematic outline of the PFV Env expression constructs. On top the outline of the wild type full-length PFV Env coding sequences are show as grey boxes with different hydrophobic sequence indicated as black boxes as explained below. The major processing sites in gp130^Env^ are marked by solid arrows. Numbers indicate amino acid positions in PFV Env. Below an outline of the individual Env expression constructs is shown. All constructs harbor a C-terminal flexible (Gly_4_Ser_1_)_3_ linker (L) and GST-tag. W denotes the conserved tryptophan residues at position 10 and 13 of the PFV Env ORF. CC: coiled-coil motif; CTRS: cytoplasmic targeting and retention signal; L: PSAP late-assembly (L)-domain motif; A: YXXLGL assembly domain motif; GR: glycine-arginine rich box; h: hydrophobic domain of the leader peptide (LP); FP: fusion peptide of the transmembrane subunit (TM); MSD: membrane-spanning domain of the TM subunit; SU: surface subunit; Y: N-glycosylation sites.

Unlike orthoretroviruses the FV Gag protein does not show the classical tripartite processing pattern into matrix (MA), capsid (CA) and nucleocapsid subunits (Figure 1A) (reviewed in [17]). Although the FV Gag protein apparently lacks a membrane targeting signal, several other sequence and structural motifs within FV Gag proteins have been characterized that have important roles for the biological function of the FV capsid protein (reviewed in [9]). These include four putative coiled-coil (CC1-4) motifs found in PFV Gag, of which only CC2 and CC3 are evolutionary conserved in many but not all FV species (Figure 1A and ref. [18]). CC2 is is required for PFV Gag-Gag interactions and is essential for capsid assembly whereas CC3 appears to mediate attachment of PFV capsids to the light chains of dynein motor proteins exploited by FVs for transport to the centrosome in target cells [19]. No function has been assigned to CC4. The first putative motif (CC1) is predicted with a low CC-forming score for FVs of great apes, but is absent in FV Gag proteins of most old word and new world monkeys as well as in the feline, bovine or equine FV Gag proteins. The putative PFV CC1 motif resides in the N-terminal part of Gag that is suggested by previous studies to harbor the poorly defined interaction domain with the CyD of the Env LP subunit [14,20-22]. For example it was demonstrated that the N-terminal 297 aa of PFV Gag, containing the essential late assembly (L) domain, are sufficient for an efficient Gag release into the supernatant when co-expressed with PFV Env [20,23]. Furthermore, small deletions within the N-terminal part of the protein abolished capsid egress and N-terminal fusion of protein tags to PFV Gag appears to interfere with viral egress in tag size dependent manner [20,22,24]. Finally, surface plasmon resonance studies using recombinant N-terminal protein fragments of feline FV (FFV) Gag (corresponding to PFV Gag aa1-180) and FFV Env peptides or recombinant protein fragments (corresponding to PFV Env aa1-28 or aa1-61) suggest a direct low affinity interaction between both proteins [14,21]. Furthermore, these studies confirm the importance of the conserved tryptophan residues with the Env LP subunit for this process. However, they failed to provide evidence for a role of the cytoplasmic targeting retention signal (CTRS) motif, responsible for capsid assembly at the centrosome, in the interaction with the viral glycoprotein [14,21,25].

Here we report a thorough investigation of the PFV Gag – Env interaction by using a pull-down assay in combination with particle release studies and single cycle replication assays using recombinant PFV vector particles. The analysis demonstrates a specific direct Gag-Env interaction that requires a minimal domain comprising amino acid 11–129 of PFV Gag. Interestingly, functional data of selected mutants of N-terminal amino acids of this minimal domain indicate that this PFV capsid structure might have additional Env-interaction-independent functions in the FV replication cycle.

In the course of this manuscript preparation and review, the X-ray structure of an N-terminal fragment of PFV Gag in complex with peptides of the PFV Env LP subunit was published [26]. In line with the results of our study this PFV Gag-Env structure demonstrates an interaction of the N-terminus of PFV Env LP subunit with PFV Gag through a hydrophobic pocket involving an N-terminal amphipathic α-helix.

## Results

### Deletion of the N-terminal 130 aa of PFV Gag abolishes particle release

**Figure 2:**
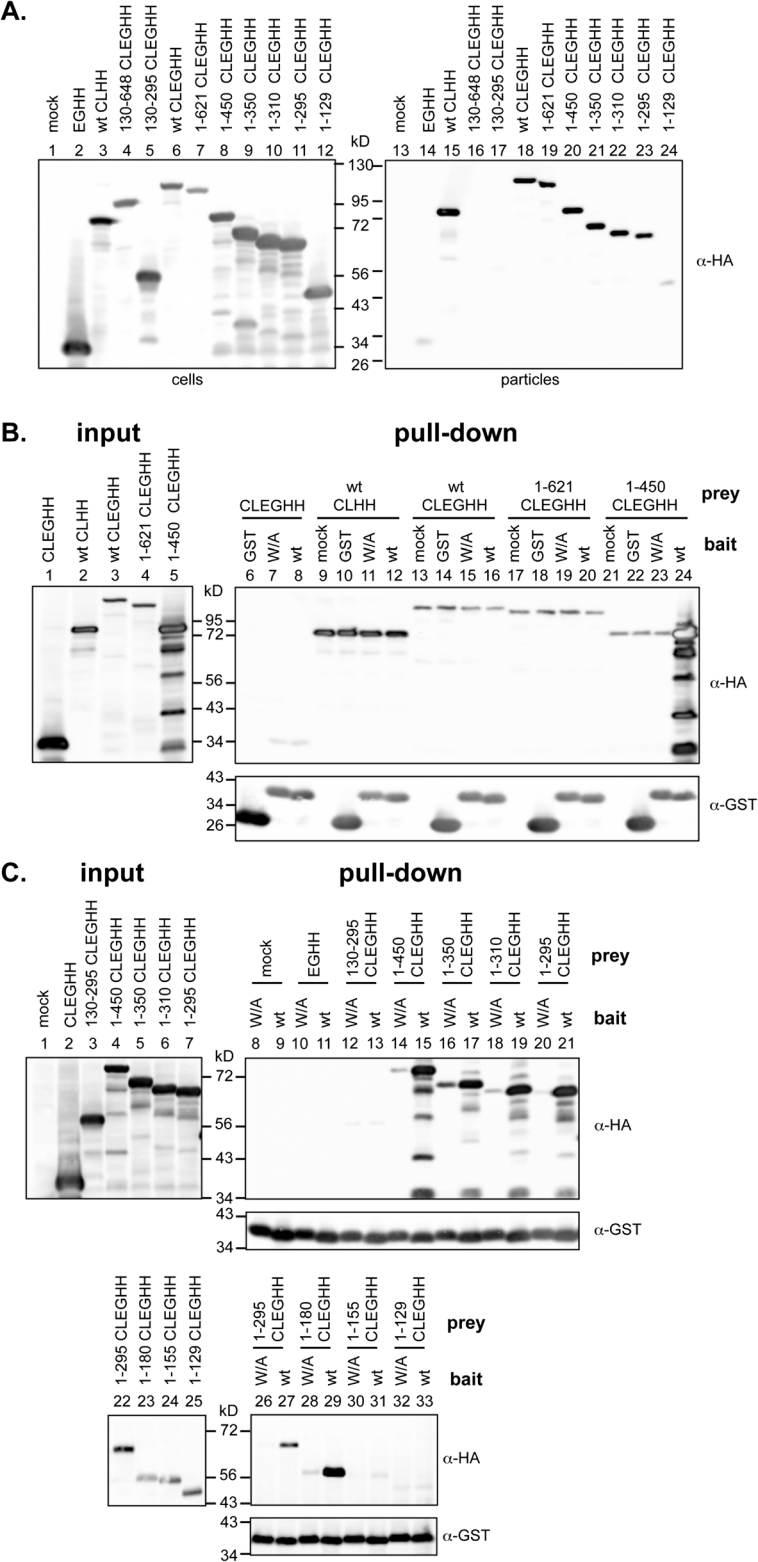
**Release and interaction characteristics of various PFV Gag – Env protein combinations.** (**A**) Representative Western blot analysis of 293T cell lysates (cells) and subtilisin-digested viral particles preparations (particles) purified by ultracentrifugation through 20% sucrose, transiently transfected with different combinations of Gag and Env expression constructs as indicated. Gag proteins were detected using a polyclonal anti-HA serum. Cells were transfected with pczHFVenvEM002 and lane 2, 14: pcziCLEGHH (EGHH); lane 3, 15: pcziPG4 CLHH (wt CLHH); lane 4, 16: pcziPG4 130–648 CLEGHH (130–648 CLEGHH); lane 5, 17: pcziPG4 130–295 CLEGHH (130–295 CLEGHH); lane 6, 18: pcziPG4 CLEGHH (wt CLEGHH); lane 7, 19: pcziPG4 1–621 CLEGHH (1–621 CLEGHH); lane 8, 20: pcziPG4 1–450 CLEGHH (1–450 CLEGHH); lane 9, 21: pcziPG4 1–350 CLEGHH (1–350 CLEGHH);; lane 10, 22: pcziPG4 1–310 CLEGHH (1–310 CLEGHH); lane 11, 23: pcziPG4 1–295 CLEGHH (1–295 CLEGHH); lane 12, 24: pcziPG4 1–129 CLEGHH (1–129 CLEGHH); or lane 1, 13: only pUC19 (mock). (**B-C**) Representative Western blot analysis of pull-down assays using mixed lysates of 293T cells transiently transfected with different GST-tagged Env or EGFP-HH-tagged Gag expression constructs as indicated. Samples of Gag expressing cell lysates corresponding to 10% of the input (input) for the pull-down assay are shown to the left, samples of proteins eluted by boiling in SDS-PAGE loading buffer from pelleted glutathione-sepharose beads (pull-down) are shown to the right. Prey (Gag and ctrls) proteins were detected using a polyclonal anti-HA serum (α-HA), bait (Env and ctrls) proteins using a polyclonal anti-GST serum (α-GST). Shown are pull-down assays combining 293T cell lysates transiently transfected with various prey or bait expression constructs as indicated. Preys: EGHH: pcziCLEGHH; wt CLHH: pcziPG4 CLHH; wt CLEGHH: pcziPG4 CLEGHH; 1–621 CLEGHH: pcziPG4 1–621 CLEGHH; 1–450 CLEGHH: pcziPG4 1–450 CLEGHH; 1–350 CLEGHH: pcziPG4 1–350 CLEGHH; 1–310 CLEGHH: pcziPG4 1–310 CLEGHH; 1–295 CLEGHH: pcziPG4 1–295 CLEGHH; 1–180 CLEGHH: pcziPG4 1–180 CLEGHH; 1–155 CLEGHH: pcziPG4 1–155 CLEGHH; 1–129 CLEGHH: pcziPG4 1–129 CLEGHH. Baits: mock: pCAGGS; GST: pCAG GST; wt: pCAG Env1-60 CLGST; W/A: pCAG Env1-60 CLGST W/A.

Previous studies have suggested, that the PFV Gag-Env interaction is mediated by amino-acids in the N-terminal region of the Gag [14,20-22]. To identify a suitable Gag construct for establishment of a eukaryotic pull-down assay to study the Gag-Env interaction and to re-examine the requirements for PFV Env-dependent particle egress, we first determined the particle release capacity of a panel of Gag truncations (Figure 1B). All PFV Gag proteins contained a C-terminal eGFP-HA tag to allow detection of the differently sized proteins in Western blot analysis with the same efficiency using tag-specific antibodies. C-terminal tagging was chosen since it was previously shown that C- but not N-terminal PFV Gag fusion proteins have wild type-like particle release features [24]. In line with this transient transfection of 293T cells with different PFV Gag packaging constructs, in combination with a full-length PFV Env packaging construct, revealed an efficient PFV Env-dependent particle egress for full-length PFV Gag proteins with either C-terminal HA- or EGFP-HA-tag (Figure 2A, lane 15, 18). Successive C-terminal deletion of PFV Gag (up to aa 295) did not significantly reduce particle release as long as the PSAP L-domain was retained (Figure 2A, lane 19–23). A truncated Gag protein comprising only the N-terminal 129 aa still supported capsid export, but at significantly reduced levels compared to all other tested C-terminal truncations containing the PSAP L-domain (Figure 2A, lane 24). In contrast, no particles were released upon deletion of aa 1–129 of either the full-length Gag protein or a protein comprising the first 295 aa from cells co-expressing Env (Figure 2A, lane 16, 17), although both contained the L-domain motif and were expressed at similar levels as Gag proteins still supporting particle egress (Figure 2A, lane 1–12).

This suggests the first 129 aa of PFV Gag to be sufficient for Env-dependent particle egress and to harbor the main determinants for interaction with the glycoprotein although a loss of Env requirement in particle release cannot be formally excluded from our experiments. However, in previous studies similar C-terminal Gag truncations were demonstrated to retain their Env-dependence in particle egress [20].

### Examining the specificity of the PFV Gag – Env interaction by a eukaryotic pull-down assay

In order to map the domains of PFV-Gag that interact with the Env-LP in further detail a GST-based pull–down assay employing a mammalian cell expression system was developed. In this assay different PFV Gag prey proteins were incubated with an Env-GST fusion protein bait, comprising residues 1–60 of Env (Env-LP-GST wt) (Figure 1C). Alternatively, a derivative (Env-LP-GST W/A) where the essential W_10_ and W_13_ of Env were substituted with alanine, just GST (GST), or mock transfected cell lysates were used as bait to examine the specificity of any interactions detected (Figure 1C).

It has been demonstrated previously that full length PFV Gag harbouring the GR-boxes is unsuitable for a pull-down due to problems associated with high background and poor specificity of co-immunoprecipitation [18]. Therefore PFV Gag fusion protein preys with different C-terminal deletions were first tested for suitability in this assay (Figure 2B). These data show that the Gag proteins extending beyond the amino-terminal 450 residues display weak but significant non-specific binding with the affinity matrix (Figure 2B, compare lanes 9–11, 13–15 and 17–19 with 12, 16 and 20). Neither nuclease pre-treatment of cell lysates nor variation of salt conditions improved the specificity of the pull down of these larger Gag baits (data not shown).

However, a Gag fragments comprising the amino-terminal 450 residues displayed strong specific binding with the wild type bait and little nonspecific binding with the controls (Figure 2B, compare lane 21–23 with 24). Therefore, various Gag fragments up to the amino-terminal 450 residues were then tested for their ability to interact with the different baits (Figure 2C). Detailed analysis of Env interaction with the different Gag-fusion protein baits indicated that the first 180 aa were sufficient for maintaining a specific Env tryptophan residue-dependent co-precipitation of Gag (Figure 2C, lane 14–21, 26–29). Gag-fusion proteins comprising the first 129 or 155 aa or the domain of aa 130–295 failed to specifically interact with Env-GST bait proteins (Figure 2C, lane 12, 13, 30–33), although they were present in the lysates at levels similar to proteins that were co-precipitated specifically (e.g. 1–180 CLEGHH) (Figure 2C, lane 3–7, 22–25). In samples where Gag proteins were co-precipitated in an Env-specific manner, additional shorter Gag forms were also detectable with the C-terminal tag specific antiserum, most prominent for the Gag 1–450 prey protein (Figure 2C, lane 8–21). The nature of these, probably N-terminally truncated, shorter forms is unclear. They might represent degradation products, which are co-precipitated with the full-length protein by Gag-Gag interactions, as they are already detectable in the cell lysates used in the pull-down assays (Figure 2C, lane 1–7).

Taken together these results suggest that in a pull-down assay the first 180 aa of PFV Gag are sufficient and domains within the first 130 aa are essential for the specific interaction with the CyD of PFV Env LP.

### Only the N-terminal 10 aa of PFV Gag are dispensable for PFV particle release

**Figure 3:**
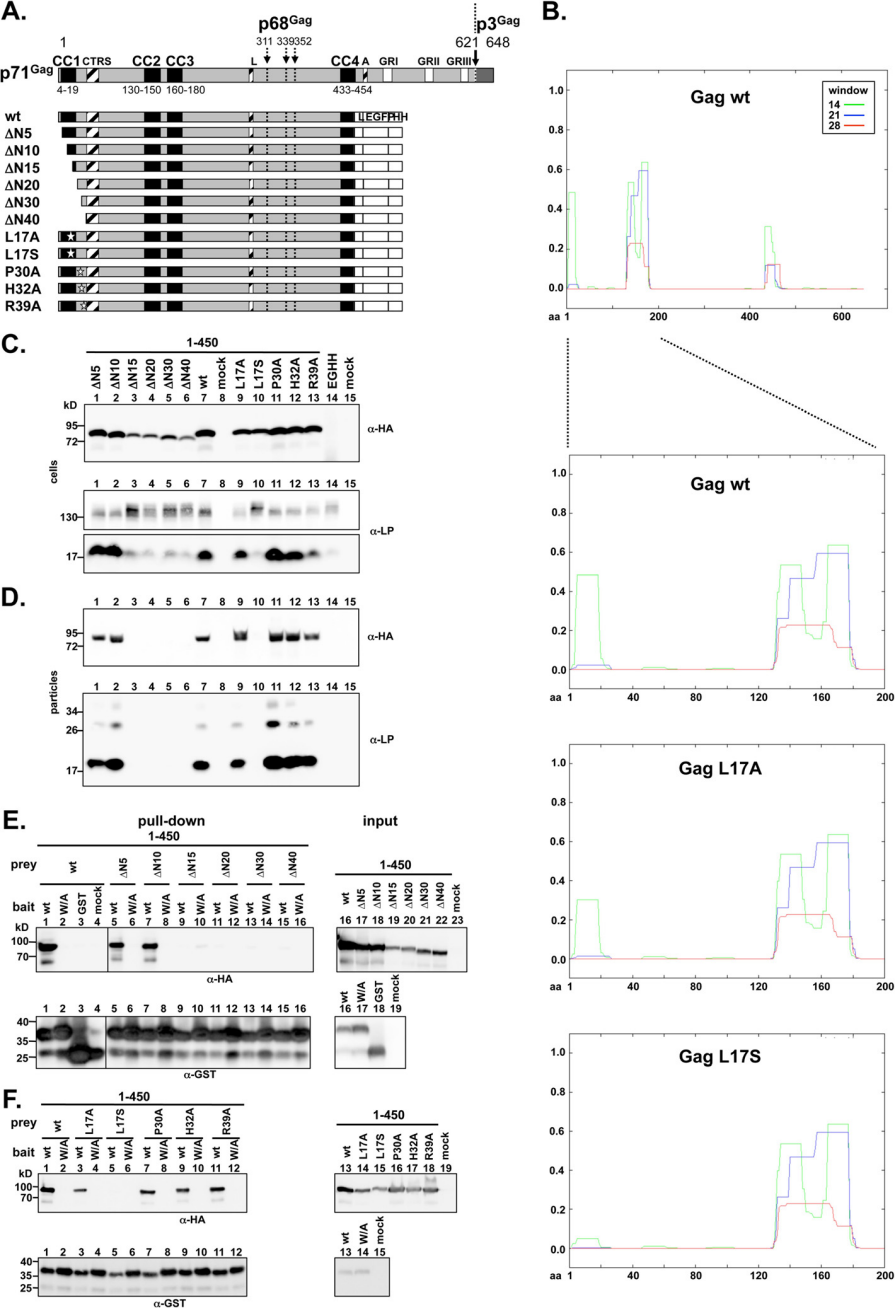
**Release and interaction characteristics of PFV Gag proteins with small N-terminal deletions or point mutations.** (**A**) Schematic outline of the PFV Gag expression constructs. On top the outline of the wild type full-length PFV Gag coding sequences are shown as grey boxes with different structural and functional domains indicated in differentially shaded boxes as described in the legend of Figure 1. (**B**) Coiled-coil domain prediction using COiLS v2 algorithm (embnet.vital-it.ch/software/coils) [34] for different PFV Gag proteins. Shown are the graphic displays of the analysis for full-length wild type protein as well as the N-terminal domains (aa 1–200) of wild type (wt), as well as mutants L_17_A (L17A) and L_17_S (L17S). (**C-D**) Representative Western blot analysis of 293T cell lysates (cells) and viral particles preparations (particles) purified by ultracentrifugation through 20% sucrose, transiently transfected with different combinations of Gag and Env expression constructs as indicated. Gag proteins were detected using a polyclonal anti-HA serum (α-HA), Env proteins using a polyclonal anti-PFV Env LP serum (α-LP). Cells were transfected with pczHFVenvEM002 and lane 1: pcziPG4 6–450 CLEGHH (∆N5); lane 2: pcziPG4 11–450 CLEGHH (∆N10); lane 3: pcziPG4 16–450 CLEGHH (∆N15); lane 4: pcziPG4 21–450 CLEGHH (∆N20); lane 5: pcziPG4 31–450 CLEGHH (∆N30); lane 6: pcziPG4 41–450 CLEGHH (∆N40); lane 7: pcziPG4 1–450 CLEGHH (wt); lane 9: pcziPG4 1–450 L17A CLEGHH (L17A); lane 10: pcziPG4 1–450 L17S CLEGHH (L17S); lane 11: pcziPG4 1–450 P30A CLEGHH (P30A); lane 12: pcziPG4 1–450 H32A CLEGHH (H32A); lane 13: pcziPG4 1–450 R39A CLEGHH (R39A); lane 14: pcziCLEGHH (EGHH); or lane 15: only pUC19 (mock). (**E-F**) Representative Western blot analysis of pull-down assays using mixed lysates of 293T cells transiently transfected with different GST-tagged Env or EGFP-HH-tagged Gag expression constructs as indicated. Samples of Gag expressing cell lysates corresponding to 10% of the input (input) for the pull-down assay are shown to the right, samples of proteins eluted by boiling in SDS-PAGE loading buffer from pelleted glutathione-sepharose beads (pull-down) are shown to the left. Prey (Gag) proteins were detected using a polyclonal anti-HA serum (α-HA), bait (Env and ctrls) proteins using a polyclonal anti-GST serum (α-GST). Shown are pull-down assays combining 293T cell lysates transiently transfected with various prey or bait expression constructs as indicated. Preys: wt: pcziPG4 1–450 CLEGHH; ∆N5: pcziPG4 6–450 CLEGHH; ; ∆N10: pcziPG4 11–450 CLEGHH; ; ∆N15: pcziPG4 16–450 CLEGHH; ; ∆N20: pcziPG4 21–450 CLEGHH; ; ∆N30: pcziPG4 31–450 CLEGHH; ; ∆N40: pcziPG4 41–450 CLEGHH; wt: pcziPG4 1–450 CLEGHH; L17A: pcziPG4 1–450 L17A CLEGHH; L17S: pcziPG4 1–450 L17S CLEGHH; P30A: pcziPG4 1–450 P30A CLEGHH; L17A: pcziPG4 1–450 H32A CLEGHH; R39A: pcziPG4 1–450 R39A CLEGHH; mock: pUC19. Baits: mock: pCAGGS; GST: pCAG GST; wt: pCAG Env1-60 CLGST; W/A: pCAG Env1-60 CLGST W/A.

To map Gag domains essential for the PFV Env interaction in further detail small successive N-terminal truncation- or selected point mutants were examined in context of the Gag 1–450 -eGFP-HA fusion protein for their ability to support capsid release upon co-expression with full-length Env (Figure 3A-D). The effects of the same Gag mutations on the interaction with the CyD of the Env LP subunit were also assessed using the pull-down assay (Figure 3E + F). Point mutations were selectively introduced into conserved amino acids within the N-terminal 40 aa that have not been functionally examined previously (Figure 3A). Furthermore, a potential contribution of aa L_17_ within the predicted CC1 previously reported to be important for particle egress [20] was assessed by introducing either a conservative (L_17_A mutant) or more deleterious (L_17_S mutant) (Figure 3A + B).

The analysis revealed that N-terminal deletions of up to 10 aa did not alter particle release, whereas egress was completely abolished by deletions of 15 aa or more (Figure 3C + D, lane 1 to 7). Most of the point mutations examined had no or only minor negative effects on Gag protein release, except for Gag L_17_S, which showed a strong reduction (Figure 3C + D, lane 7 to 13). Notably, Gag proteins with point mutations at L_17_ or deletions of 15 aa or more showed reduced steady state protein levels in cell lysate samples (Figure 3C, lane 3 to 6, 9, 10). Furthermore, in cell lysate samples of all Gag constructs that did not support particle release a clearly diminished Env processing was observed (Figure 3C, lane 3–6, 10). This is in line with previous observations demonstrating that co-expression of wild type Gag enhances Env cell surface expression as well as Env precursor processing by furin-like proteases in the late Golgi compartment [15,27].

In agreement with the particle release analysis data, Gag proteins with N-terminal deletions of more than 10 aa did not co-precipitate with the CyD of the Env LP subunit (Figure 3E). Furthermore, the pull-down assay showed an interaction for all Gag point mutants except L_17_S (Figure 3F). Thus, the N-terminal 10 aa of PFV Gag, which includes the N-terminal third of the predicted CC1 motif (aa 4–19) appears to be dispensable for the interaction with the Env LP subunit and for particle release. In contrast, larger N-terminal deletions extending further into the putative CC1 motif sequence as well as a point mutation within CC1 interfering with the structural integrity are detrimental to these processes.

### In vitro interaction analysis of recombinant PFV Gag and Env protein fragments

**Figure 4:**
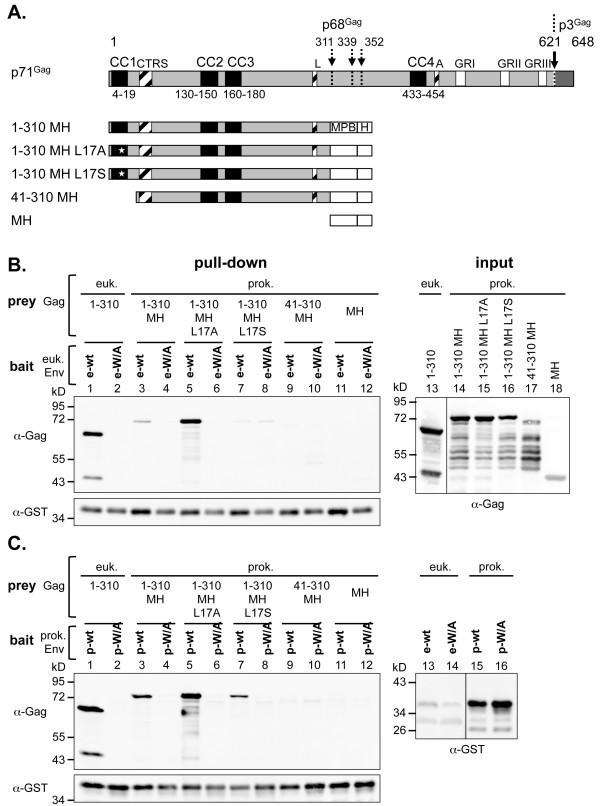
**Gag-Env interaction analysis using different combinations of prokaryotic and eukaryotic expressed proteins.** (**A**) Schematic outline of the prokaryotic PFV Gag expression constructs. On top the outline of the wild type full-length PFV Gag coding sequences are shown as grey boxes with different structural and functional domains indicated as in the legend of Figure 1. (**B-C**) Representative Western blot analysis of pull-down assays using various combination of lysates of 293T cells (euk.) containing different GST-tagged Env or EGFP-HH-tagged Gag proteins or affinity purified proteins expressed in *E. coli* (prok.) as indicated. Samples of Gag expressing cell lysates corresponding to 10% of the input (input) for the pull-down assay are shown to the right, samples of proteins eluted by boiling in SDS-PAGE loading buffer from pelleted glutathione-sepharose beads (pull-down) are shown to the left. Prey (Gag and ctrls) proteins were detected using polyclonal antibodies specific for PFV Gag (α-Gag), bait (Env) proteins using a polyclonal anti-GST serum (α-GST). Preys: 1–310: pcziPG4 1–310 CLEGHH; 1–310 MH: pET11a Gag 1–310 MH; 1–310 MH L17A: pET11a Gag 1–310 MH L17A; 1–310 MH L17S: pET11a Gag 1–310 MH L17S; 41–310 MH: pET11a Gag 41–310 MH; MH: pET11a MH. Baits: e-wt: pCAG Env1-60 CLGST; e-W/A: pCAG Env1-60 CLGST W/A; p-wt: pET11a Env1-60 CLGST; p-W/A: pET11a Env1-60 CLGST W/A.

The results presented above strongly indicate a specific interaction between the N-terminal domains of PFV Gag and Env to be essential for particle egress. Nevertheless, it remained unclear whether proteins interacted directly or required additional factors. Therefore, fragments of both wild type and selected mutants of PFV Gag and Env were recombinantly expressed in *E.coli* and affinity-purified proteins used for *in vitro* interaction analysis studies (Figure 4A). Notably, of the different Gag variants tested, constructs spanning the first 310 aa of PFV Gag with a C-terminal MBP fusion tag yielded the highest amounts of soluble recombinant protein (data not shown).

Subsequently, interaction analysis assays were performed by incubation of one interaction partner of prokaryotic origin with lysates of transiently transfected 293T cells containing the respective other partner or by co-incubation of purified recombinant proteins of both interaction partners (Figure 4B + C). The results demonstrate that eukaryotic PFV Env-LP-GST fusion proteins can interact with recombinant PFV Gag proteins expressed in *E.coli* with a similar specificity as observed for respective eukaryotic cell-derived protein (Figure 4B, lane 1–4). The specific interaction was abolished by mutation of the conserved tryptophan residues in Env-LP as well as by deletion of the N-terminal 40 aa of Gag or mutation of leucine at position 17 into serine (Figure 4B, lane 1–12). Furthermore, an almost identical result was obtained when using PFV Env-LP-GST fusion proteins expressed in *E.coli*. Recombinant PFV Env-LP-GST was able to co-precipitate PFV Gag expressed in mammalian cells in a tryptophan-residue-dependent manner (Figure 4C, lane 1, 2). In addition, recombinant PFV Gag showed a specific co-precipitation, that was dependent on the presence of the first 40 aa of PFV Gag (Figure 4C, lane 3–12).

A few differences were noted. First, wild type prokaryotic PFV Gag appeared to interact less strongly with the Env-LP-GST bait proteins than the eukaryotic Gag protein when using similar amount of prey protein (Figure 4B + C, lane 1–4). This phenomenon was more pronounced for bait proteins of eukaryotic than of prokaryotic origin. Second, the recombinant prokaryotic Gag L_17_A prey seemed to be pulled down more efficiently than the respective wild type protein at similar amounts of input (Figure 4B + C, lane 3–6). Third, unlike mammalian cell-derived PFV Env-LP-GST, the equivalent recombinant prokaryotic protein specifically co-precipitated the recombinant Gag L_17_A and the Gag L_17_S mutant proteins (Figure 4C, lane 5–8). However, the Gag L_17_S mutant was co-precipitated to a much lower extent than the L_17_A mutant.

In summary, a specific Gag-Env interaction requiring conserved tryptophan residues in Env-LP can be observed with purified *E. coli* expressed protein fragments, indicating that the interaction between Gag and Env is direct and does not require additional co-factors. Moreover, the interaction most probably relies on the N-terminal sequences as it requires the first 40 aa of PFV Gag and is abolished the Gag L_17_S mutation.

### Particle egress and infectivity supported by full-length PFV Gag proteins with N-terminal deletions or point mutations

**Figure 5:**
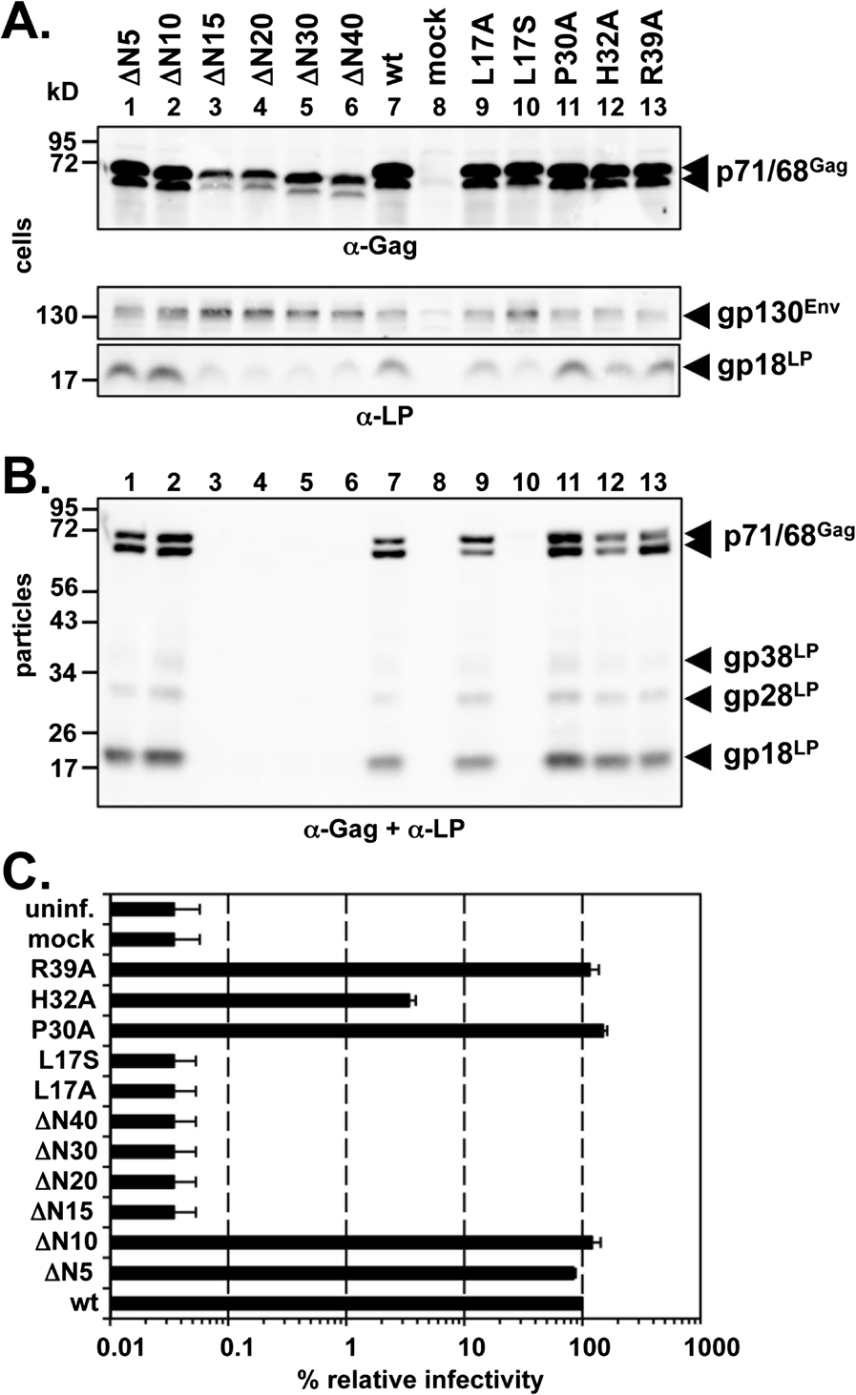
**Single-round infection analysis phenotype of PFV Gag proteins with small N-terminal deletions and point mutations.** 293T cells were co-transfected with equal amounts of PFV transfer vector puc2MD9, Env packaging vector pcziPFVenvEM002, Pol packaging vector pcziPol and various Gag packaging constructs (wt: pcziPG4; ∆N5: pcziPG4 6–648; ∆N10: pcziPG4 11–648; ∆N15: pcziPG4 16–648; ∆N20: pcziPG4 21–648; ∆N30: pcziPG4 31–648; ∆N40: pcziPG4 41–648; L17A: pcziPG4 L17A; L17S: pcziPG4 L17S; P30A: pcziPG4 P30A; H32A: pcziPG4 H32A; R39A: pcziPG4 R39A) or only with pUC19 (mock) as indicated. Western blot analysis of (**A**) cell lysates (cells) and (**B**) pelleted viral supernatants (particles) with polyclonal antibodies specific for PFV Gag (α-Gag), and PFV Env-LP (α-LP). The identity of the individual proteins is indicated on the right. **C**) Relative infectivity of extracellular 293T cell culture supernatants using an eGFP marker gene transfer assay was determined 3 days post infection. The values obtained using the wild type Gag packaging vector pcziPG4 were arbitrarily set to 100%. Absolute titers of these plain supernatants were 5.3 ± 3.5 × 10^5^ EGFP ffu/ml. Means and standard deviations of three independent experiments are shown.

Identical N-terminal small deletion and point mutations of respective full-length PFV Gag packaging constructs in context of a replication-defective PFV vector system were examined for their effect in single-round infections (Figure 5). In general the expression patterns and particle release characteristics of the full-length Gag mutants were very similar to their respective protein fragments spanning the first 450 aa that were employed in the pull-down assays shown above (compare Figures 3D and 5B). Only N-terminal deletions larger than 10 aa, as well as the L_17_S point mutant, completely abolished particle egress (Figure 5B, lane 3–6, 10). The L_17_A Gag mutant consistently altered the p71^Gag^ to p68^Gag^ ratio in particle samples and the H_32_A Gag point mutant in some experiments showed a reduced particle release (Figure 5B, lane 9, 12). No significant differences in particle associated LP- and SU-subunit levels of these two mutant particles in comparison to wild type were detectable (Figure 5B, lane 9–12; and data not shown).

The analysis of the infectivity, detectable in the supernatant of 293T cells transfected with the different Gag packaging constructs, mirrored the particle release characteristics of the individual samples quite well (Figure 5C). Exceptions were again the L_17_A Gag mutant, for which no infectivity was detectable although wild type-like particle egress was observed, and the H32A Gag mutant, which displayed a 25-fold reduced infectivity although particle release was only slightly reduced in some experiments (Figure 5B + C).

These data show that in the full-length protein context the first 10 aa of PFV Gag are also dispensable for particle release and productive infection of target cells. In contrast, the sequence between amino acid 11–40 appears to be critical for particle egress. Furthermore L_17_ appears to have an additional particle release-independent function critical for particle infectivity that is impaired by mutation to alanine.

## Discussion

Although specific interactions between capsid and glycoproteins have been reported for other retroviruses, FVs are unique as their specific Gag – Env interaction is a prerequisite for budding of viral particles (reviewed in [1,8]). The major interaction domain in the FV Env protein is well defined. It spans a region of about 20 aa at the N-terminus of the LP subunit CyD that contains two essential conserved tryptophan residues [13,14]. In contrast, regions of FV Gag that are essential and sufficient for the interaction with Env are less well characterized. Previous studies suggested that the major interaction determinants are found in the N-terminal regions of FV Gag [14,20-22]. Furthermore, while this manuscript was under review the X-ray structure of an N-terminal fragment of PFV Gag in complex with a peptide of the PFV Env LP subunit was determined confirming that residues within the Gag CC1 motif are important for PFV Gag-Env interaction [26].

Our particle release studies suggest aa 11 to 129 of PFV Gag is sufficient for the specific interaction with PFV Env enabling budding of virions, although longer constructs containing the L-domain motif were secreted more efficiently. Furthermore, our data indicate that residues 11–40 of PFV Gag and their structural integrity are essential for the interaction with PFV Env in mammalian cells. In support of this notion, the recent Gag NTD-Env LP NTD structure shows that residues V_14_ to R_24_ comprise an amphipathic α-helix that forms a major part of the Env-LP interaction site [26].

The main observations of our particle release studies were also confirmed by the mammalian pull-down assays. However, the minimal PFV Gag proteins that showed an efficient and specific interaction with the CyD of PFV Env LP necessarily included the predicted CC2 (aa 130–150) and (CC3 (aa 160–180) regions in PFV Gag. CC2 and CC3, which are conserved in Gag proteins of many but not all FV species, have been reported to have essential function in intermolecular PFV Gag interactions (CC2) and association of PFV Gag with cellular proteins (CC3) [18,19]. The direct participation of CC2 and CC3 in the interaction with Env appears unlikely because unlike CC1 they are not required in the particle release assays. However, their requirement for efficient Env co-precipitation suggests an influence of the oligomeric state of Gag on the interaction with Env. N-terminal PFV Gag proteins lacking these CC domains are unlikely to form higher oligomeric structures, although this was not directly addressed in this study. In line with this notion in the Gag-Env crystal structure, aa R_140_ to S_179_, comprising most of CC2 and CC3, form a single continuous α-helix and are organized in a single CC domain that makes up the majority of the Gag dimer interface [26].

Results from our interaction studies employing *E. coli* produced PFV Gag and Env protein fragments also support the observations of the Gag-Env crystal structure and a previous report for FFV that suggested a direct interaction of both viral proteins, without a requirement for posttranslational modification or additional cellular proteins [14,26]. Importantly, as in the mammalian pull-down assay the presence of the N-terminal 40 aa of PFV Gag was essential for the interaction with Env when using recombinant protein fragments. The reason and potential functional significance of an apparent difference in the interaction strength of wt Gag-derived protein fragment of prokaryotic to eukaryotic origin that were noted are currently unclear. They might be the result of different C-terminal tags on both types of proteins used (EGFP-His-HA-tag for eukaryotic and MBP-His-tag for prokaryotic proteins).

A major difference in our pull-down assays performed with recombinant bait and prey proteins of different origins was a residual binding activity of prokaryotic-derived PFV Gag L_17_S to prokaryotic- but not mammalian-derived GST-Env LP. This might indicate that multiple residues of the N-terminal Gag interaction domain contribute to the interaction with the Env LP subunit. Alternatively, the Gag L_17_S mutant protein might fold differently in eukaryotes than in prokaryotes. In line with these results Goldstone and colleagues observed only a 2-fold reduction in binding affinity for prokaryotic-derived PFV Gag L_17_S to Env peptides [26].

Finally, the major phenotypes of the different Gag mutants observed in the release- and pull-down assays using PFV Gag fragments (dispensability of the N-terminal 10 aa, importance of adjacent sequences and L_17_ but not other conserved aa in the N-terminus) could be confirmed in context of the full-length proteins using a replication-deficient PFV vector system and single cycle infectivity assays. Very interesting was again the phenotype of the Gag L_17_A mutation that unlike the L_17_S mutation should retain the putative helical structure. In contrast to the L_17_S mutant L_17_A PFV Gag displayed wild type-like release characteristics, but no infectivity was detectable (>3000-fold reduced). This strongly implies an additional important function of the Gag NTD domain that is independent of its potential structural role for the Gag-Env interaction. Preliminary results indicate that this mutant fails to undergo proper intra-particle reverse transcription (data not shown).

Our observations are in line with previous studies that reported characterization of determinants in Gag and Env essential for the unique budding strategy of FVs [13,14,20-22]. Surprisingly we determined a clear dispensability of the N-terminal 10 aa of PFV Gag for FV replication in all assays including PFV vector particle infectivity. A previous study had suggested that the immediate N-terminus of PFV Gag might be involved in the Env interaction since specific mutations within the first 10 aa strongly interfered with capsid morphology and infectivity [22]. In light of our results the phenotypes of these mutants described previously may be a consequence of changes in the overall structure of the capsid rather than a direct contribution of the N-terminal 10 aa to the interaction with Env. Consistent with our finding of the dispensability of the N-terminal 10 aa of PFV Gag for viral replication is the observation that in the recently solved Gag – Env fragment structure residues preceding PFV Gag E9 are disordered in the crystal and that they seem to be non-essential for the interaction with the PFV Env LP peptides [26].

## Conclusion

In summary, we have characterized the essential determinants in PFV Gag required for the specific interaction with PFV Env. Whereas the Gag L-domain is needed for efficient release, Gag aa 10–129 are sufficient for the interaction with Env. C-terminal sequences, but not the complete sequences of a predicted N-terminal coiled-coil domain (CC1) in PFV Gag are essential for PFV egress if not directly being involved in binding the CyD of the Env LP subunit. Furthermore, our results suggest that residues within this domain of PFV Gag have additional functions for further steps in the viral replication cycle of FVs.

## Methods

### Cell lines and culture conditions

The human kidney cell line 293T [28] and the human fibrosarcoma cell line HT1080 [29] were cultivated at 37°C and 5% CO_2_ in Dulbecco’s modified Eagle medium (DMEM) supplemented with 10% heat-inactivated fetal calf serum and antibiotics.

### Recombinant DNAs and plasmid expression constructs

The PFV 4-component vector system based on authentic PFV genomic ORFs and consisting of the packaging plasmids consisting of pcziPG4 (PFV Gag wt), pcziPol (PFV Pol wt), pczHFVenvEM002 (PFV Env wt) and the enhanced green fluorescent protein (EGFP) expressing transfer vector puc2MD9 used for production of PFV wt particles was described previously [24,30]. The coding regions of the eukaryotic expressions constructs for Gag proteins with N- or C-terminal deletions, point mutations and/or C-terminal eGFP-HA tags shown schematically in Figures 1B and 3A are based on the parental pcziPG4 packaging construct. PcziPG4 CLHH contains a C-terminal (Gly_4_Ser_1_)_3_ linker (CL) and Hexa-His and HA-tags (HH), whereas pcziPG4 CLEGHH has the eGFP ORF inserted after the (Gly_4_Ser_1_)_3_ linker sequence. Furthermore, several variants of the pcziPG4 CLEGHH vector with C-terminal and/or N-terminal deletions of different length were generated. In general the PFV Gag aa retained in the ORF are given in the name, e.g. pcziPG4 130–295 CLEGHH expresses a PFV Gag protein encoding aa 130–295 and C-terminal CLEGHH tag. For the point mutants the amino acid, their natural position in the full-length protein, and the amino acid for which is was replaced are given in the name, e.g. pcziPG4 1–450 CLEGHH L17A encodes a Gag protein spanning the first 450 aa of PFV Gag with C-terminal CLEGHH tag, which has leucine 17 changed to alanine. The coding regions of the eukaryotic expression constructs for the cytoplasmic domain (aa 1–60) of PFV Env LP subunit with a C-terminal (Gly_4_Ser_1_)_3_ linker and GST ORF in frame are shown schematically in Figure 1C, and are based on the pCAGGS backbone. pCAG Env1-60 CLGST contains aa 1–60 of the wt PFV Env ORF, its variant pCAG Env1-60 CLGST W/A has the tryptophan residues at position 10 and 13 changed to alanine, and pCAG GST harbors only the (Gly_4_Ser_1_)_3_ linker and GST ORF in frame. The prokaryotic expression constructs for the wild type (pET11a Env1-60 CLGSTh) or W_10,13_A mutant (pET11a Env1-60 CLGSTh W/A) cytoplasmic domain PFV Env LP subunit, which are based on the pET11 vector backbone (Novagen), contain the identical coding regions as their eukaryotic counterparts except for an additional C-terminal in frame deca-His-tag. The prokaryotic expression constructs for PFV Gag shown schematically in Figure 4A are based on pET11a and contain PFV Gag coding sequences of variable length as indicated by their name and a C-terminal in frame maltose-binding-protein (MBP) ORF and hexa-His-tag. For example pET11a Gag 1–310 MH L17A contains aa 1–310 of PFV Gag with leucine 17 changed to alanine.

All constructs were created by standard PCR and cloning methods. Further details on the cloning procedures and the individual plasmids are available on request.

### Viral supernatant production

FV vector particles were produced as described previously [31]. Briefly, the individual plasmids of the PFV 4-component PFV vector system were transiently co-transfected into 293T cells using polyethyleneimine (PEI) in 10cm dishes. 293T cells were co-transfected with different combinations of PFV Gag and glycoprotein expression constructs as indicated. In experiments examining the infectivity of viral supernatants the PFV Gag and Env expression constructs were co-transfected together with PFV Pol packaging constructs (pcziPol) and a transfer vector (puc2MD9) at the ratio of 1:1:1:1 as indicated. If necessary, empty pUC19 plasmid was used in all experiments as stuffer to keep the total DNA amount of transfected DNA constant 15 μg total DNA per dish. Cell-free viral vector supernatant was harvested using 0.45 μm sterile filters (10 cm dish).

### Analysis of viral infectivity

Infectivity of viral supernatants produced was determined by a flow cytometric based EGFP marker gene transfer assay as described previously [24]. HT1080 cells were seeded at the density of 2 × 10^4^ cells/well in 12-well plates 16–24 h prior to transduction. Target cells were incubated with 1 ml of cell free viral supernatant generated by transient transfection as described above and 10-fold serial dilutions thereof for 4–6 h before replacement with normal growth medium. The percentage of EGFP marker gene expressing cell was determined 72 h post infection (pi) by flow cytometry using a FACS Calibur (Becton Dickinson). All transduction experiments were performed at least three times and, in each independent experiment, the titers obtained with the untagged wild-type viruses were arbitrarily set to 100% and those of the other samples expressed as values relative to the wt control, as described previously [32].

### Western blot analysis and antisera

Cellular lysates for protein expression analysis were prepared by washing the transfected 293T cells in 10 cm dishes with PBS and following 20 min of incubation with 600 μl cell-lysis buffer (10 mM Tris/HCl pH 8.0, 140 mM NaCl, 0.025% NaN_3_, 1% Triton-100) on ice. Subsequently, cell lysates were scraped off the cell culture dishes using a rubber policeman and centrifuged through a QIAshredder (QIAGEN) to shear genomic DNA. After rinsing the QIAshredder with 600 μl 2× PPPC (100 mM Tris–HCl; pH 6.8, 24% Glycerol, 8% SDS, 0.02% Coomassie Brilliant Blue G-250, 2% ß-Mercaptoethanol) the combined flow through were boiled at 95°C for 10 min and used directly for SDS-polyacrylamide gel electrophoresis or stored at −20°C until further use.

For analysis of viral particle-associated protein composition cell-free supernatant of transfected 293T cells (10cm dish) was harvested using a syringe and a 0.45 μm pore size sterile filter. Viral particles were concentrated by ultracentrifugation at 25,000 rpm, 4°C in SW32 (76,755 g) or SW40 (78,925 g) rotors through a 20% sucrose cushion. Viral pellets were resuspended in 50 μl PBS and 50 μl 2× PPPC was added. After boiling for 10 min at 95°C the samples were used directly for SDS-polyacrylamide gel electrophoresis (SDS-PAGE) or stored at −20°C.

Cell or viral particle protein samples were separated by SDS-PAGE using a 7.5% polyacrylamide gel and analyzed by immunoblotting as described previously [13]. Polyclonal rabbit antisera specific for PFV Gag [33], for the PFV Env LP subunit [13], or for mCherry (399C) [24] and a polyclonal goat antisera specific for the influenza hemagglutinine tag (BETHYL Cat No A190-107A) were used. The gluthatione transferase (GST)-specific (4DF2) antiserum was produced by immunization of rabbits with recombinant proteins purified as described below. After incubation with a suitable horseradish peroxidase (HRP)-conjugated secondary antibody, the blots were developed with Immobilon Western HRP substrate. The chemiluminescence signal was digitally recorded using a LAS-3000 imager (Fuji).

### Recombinant protein expression

Prokaryotic expression constructs were generated by insertion of ORFs encoding C-terminally decaHis tagged PFV Gag-MBP (maltose binding protein) [pET11a PFVGag1-310-MH], PFV Env LP-GST [pET11a PFVEnvLP1-60-GSTcHis), MBP [pET11a MH] or GST [pET11a GSTcHis] fusion proteins into pET11 (Novagen). The soluble fusion proteins, expressed in *Escherichia coli* BL21(DE3) cultures after overnight induction with 0.5 mM isopropylthiogalactopyranoside (IPTG) and cultivation at 14°C were affinity purified using His-Bind resin (Novagen) according to the manufacturer’s instructions. Briefly, bacterial pellets of 1 liter cultures were resuspended in 15 ml cold lysis buffer (50 mM Tris–HCl, 150 mM NaCl [for Gag 500 mM], 1% Triton X-100, 5% Glycerol, 1% Triton X-100, 5% Glycerol, protease inhibitor cocktail [Roche Complete]. Subsequently lysozyme, sodium-deoxycholate and DNAseI were added to final concentrations of 2 mg/ml, 0.125%, and 10 μg/ml, respectively. Bacteria were lysed on ice by sonication (5 cycles of 30 sec each, Bandelin Sonoplus HD70, Cycle 50%, Power 50%). Bacterial debris was pelleted by centrifugation at 19,000 rpm (43,500xg), 4°C for 45 min and the supernatant was sterile filtrated (0.45 μm pore size) prior to loading onto the affinity column. For affinity purification His-Bind-Resin columns (Novagen) with 3 ml bed volume were used. Columns were washed with 5 volumes ddH20, activated with 2 volumes 0.1M NiSO4 solution followed by washing with 5 volumes washing buffer (lysis buffer with 5 mM Imidazol). After loading of the bacterial lysate solution and washing with 5 volumes wash buffer bound proteins were eluted with 1 ml aliquots of lysis buffer containing increasing amounts of imidazole (100 mM, 200 mM, 300 mM, 400 mM, 500 mM). Elutions containing the largest amounts were pooled and their concentration determined by SDS-PAGE using a BSA standard (0.125 to 2 mg/ml) and densitometric quantitation of the recombinant protein bands. Recombinant proteins were stored in 50% Glycerol at −80°C until further use.

### GST pull-down assay

For the GST pull-down assay eukaryotic Gag prey and Env bait proteins were obtained by transient transfection of separate 293T cultures using polyethyleneimine (PEI) as indicated above. Forty-eight hours post transfection cell lysates were obtained by washing the transfected 293T cells in 10 cm dishes with PBS and following 10 min of incubation with 1 ml pull-down-lysis buffer (50 mM Tris/HCl pH 7.4, 500 mM NaCl, 5 mM EDTA, 1 mM DTT, 5% Glycerol, 1% Triton-100) at room temperature. Subsequently, cell lysates were scraped off the cell culture dishes using a rubber policeman, transferred to 1.5 ml Eppendorf tubes and incubated for another 20 min at 4°C on a overhead-shaker. Precleared lysates were generated by pelleting of insoluble components by centrifugation in a precooled table top centrifuge for 6 min at 13,000 rpm (15,500×g), 4°C. Following transfer of the supernatant to a fresh tube the lysate was used immediately. Samples of the lysates were mixed with 2× PPPC and used as input control for SDS-PAGE and Western blot analysis.

For each pull-down sample 15 μl of Gluthatione-sepharose-beads (GE Healthcare, 75% slurry) were used. Prior to incubation with the cell lysates or recombinant proteins the combined sepharose beads were washed three times by incubation in 1 ml cold pull-down wash buffer (50 mM Tris/HCl pH 7.4, 150 mM NaCl, 5 mM EDTA, 1 mM DTT, 5% Glycerol, 0,1% Triton-100) for 1 min and subsequent pelleting by centrifugatio for 1 min at 13,000 rpm (15,500×g), 4°C. After the last wash the combined sepharose beads were resuspend in 80 μl cold pull-down wash buffer per sample and immediately aliquoted into fresh individual 1.5 ml tubes and stored on ice.

For pull-down assays each 450 μl of precleared 293T lysate containing bait or prey proteins were combined in a 1.5 ml tube containing 80 μl of activated Gluthatione-sepharose beads on ice. When using recombinant proteins bait (5 μg) and prey (200 μg) proteins were combined in a total volume of 900 μl cold pull-down-assay buffer. Following mixing of bait and prey samples as indicated, the mixture was incubated for 2 h at 4°C on a overhead-shaker. Subsequently, protein complexes bound to the sepharose beads were pelleted by centrifugation for 20 sec at 13,000 rpm (15,500×g), 4°C resuspended in 1 ml cold pull-down wash buffer and incubated for 2 min at 4°C on overhead-shaker. This washing procedure was repeated two times before resuspending the sepharose bead pellet in 100 μl 2× PPPC. Proteins were eluted by incubating the samples at 95°C for 10 min, followed by pelleting of the sepharose beads for 1 min at 13,000 rpm (15,500×g), room temperature and transfer of the supernatant to a fresh tube. Samples were used immediately for SDS-PAGE and Western blot analysis as described above or stored at −20°C until further use.

## Competing interests

The authors declare that they have no competing interests.

## Authors’ contributions

DL conceived and coordinated the study. JR, AS, AG, MR and AG performed the experiments or generated essential materials used in the study. JR, AS and DL were involved in data interpretation. DL wrote the manuscript. All authors read and approved the final manuscript.
